# Advancing In Vivo Chimeric Antigen Receptor T‐Cell Engineering to Accelerate Clinical Translation

**DOI:** 10.1002/mco2.71030

**Published:** 2026-09-27

**Authors:** Zhenxin Bai, Ming Yi, Shengtao Hu, Dixuan Xue, Luxin Wei, Lifeng Zhang, Zhijun Dai

**Affiliations:** ^1^ Department of General Surgery The Fourth Affiliated Hospital of Soochow University (Suzhou Dushu Lake Hospital) Suzhou China; ^2^ Department of Breast Surgery First Affiliated Hospital School of Medicine Zhejiang University Hangzhou China

**Keywords:** delivery systems, in vivo chimeric antigen receptor T‐cell, translational strategies, tumor immunology

## Abstract

Chimeric antigen receptor T‐cell (CAR‐T) therapy is a transformative tumor immunotherapy that redirects autologous T cells to eliminate malignant cells. However, its broader clinical translation is constrained by complex and costly ex vivo manufacturing, variable product quality, and limited control over in vivo activity. In vivo CAR‐T engineering enables direct T‐cell programming in the body, reducing reliance on ex vivo manufacturing while improving T‐cell fitness and antitumor efficacy. This approach also enables flexible dosing and may obviate lymphodepletion. This review first summarizes recent advances in in vivo CAR‐T engineering and the evolution of CAR architectures. We then examine viral and non‐viral delivery systems, including lentiviral vectors (LVs), adeno‐associated virus (AAV) vectors, lipid nanoparticles (LNPs), polymeric nanoparticles (PNPs), and emerging platforms, highlighting their distinct advantages and limitations. We further evaluate strategies to facilitate clinical translation, focusing on safety and efficacy. Finally, we discuss emerging opportunities enabled by biomaterials and artificial intelligence to improve scalability and accessibility while broadening therapeutic applications. This review provides a framework for understanding in vivo CAR‐T engineering and highlights key strategies for overcoming translational barriers and advancing next‐generation CAR‐T therapies.

## Graphical abstract

In vivo CAR‐T cell generation directly engineers endogenous T cells using pre‐manufactured gene delivery vectors that target T cells and deliver CAR‐encoding genetic material. The resulting CAR‐T cells expand in vivo and mediate antigen‐specific cytotoxicity. Emerging applications span hematological malignancies, solid tumors, autoimmune diseases, myocardial fibrosis, and infectious diseases.

## 1 Introduction

Tumors remain a leading global public health challenge, with rising incidence and mortality and imposing a considerable societal and economic burden [1]. Although recent therapeutic innovations have expanded options beyond traditional surgery and radiotherapy/chemotherapy to more precise targeted therapies, clinical efficacy in advanced or refractory malignancies—both hematologic and solid—often plateaus [2]. Against this backdrop, breakthroughs in immunotherapy have opened fundamentally new avenues for tumor treatment [3, 4]. Contemporary tumor immunotherapy encompasses immune checkpoint blockade, antibody‐drug conjugates, tumor vaccines, and adoptive cellular therapies [5, 6, 7, 8, 9, 10, 11, 12]. Among these therapeutic strategies, chimeric antigen receptor T‐cell (CAR‐T) therapy has demonstrated remarkable clinical efficacy, resulting in its rapid adoption as a cornerstone in tumor immunotherapy [13].

The fundamental principle of CAR‐T therapy is to genetically reprogram a patient's autologous T cells to express chimeric antigen receptor (CAR) molecules capable of recognizing tumor‐associated antigens, thereby generating a “living drug” with precise and potent tumor‐killing activity [14, 15]. A typical CAR construct consists of an extracellular antigen‐binding domain—usually a single‐chain variable fragment (scFv)—followed by a hinge region, a transmembrane domain, and intracellular signaling components, including CD3ζ and co‐stimulatory elements like CD28 or 4‐1BB [16, 17]. A landmark achievement in 2017 was the approval of the first CD19‐targeted CAR‐T therapy for acute B‐lymphoblastic leukemia [18]. Since then, the clinical use of CAR‐T therapy has expanded significantly, showing impressive effectiveness in treating B‐cell lymphoma, multiple myeloma, and various other hematologic cancers [19, 20, 21]. These successes have provided many patients with the possibility of durable remission and, in some cases, potential cure [22, 23, 24].

Traditional CAR‐T therapy depends on a highly intricate in vitro manufacturing pipeline [25]. T cells are initially extracted from the patient's peripheral blood [26], then genetically modified ex vivo using either viral vectors, such as lentiviral or retroviral systems, or non‐viral methods, including electroporation and transposon‐mediated delivery [27]. These engineered cells are then expanded on a large scale before being reinfused into the patient [28, 29]. This multi‐step, patient‐specific workflow underscores the central bottlenecks of conventional CAR‐T therapy, which cluster around three key domains: manufacturing complexity, cellular product quality, and dynamic control. The production process is notably laborious and time‐intensive [30], associated with exceptionally high costs [31, 32], and yields a heterogeneous cellular product [33, 34]. Furthermore, once reinfused, CAR‐T cells are difficult to regulate in vivo and can provoke severe, and occasionally life‐threatening, toxicities [35, 36]. Although CAR‐T therapy has produced groundbreaking clinical advances in hematologic malignancies [37, 38, 39], translating this success to solid tumors remains a substantial challenge [40].

Optimizing the accessibility and therapeutic specificity of CAR‐T therapy necessitates dynamic and tight control of both CAR‐T cells and the surrounding tumor microenvironment (TME) [41, 42, 43]. Yet such fine‐tuned modulation is difficult to achieve within the constraints of static, ex vivo manufacturing workflows [44, 45]. These limitations have catalyzed growing interest in an emerging paradigm—in vivo CAR‐T. This strategy effectively shifts the CAR‐T “manufacturing plant” from external bioreactors to the patient's own body. In vivo CAR‐T strategies employ delivery vectors carrying CAR‐encoding DNA, messenger RNA (mRNA), or related constructs, which are administered systemically or locally [46, 47]. Through intrinsic tropism or engineered targeting features, these vectors selectively engage T cells—or other immune cell populations—within the body [48], enabling direct gene transfer and CAR expression in situ [49]. As a result, functional CAR‐T cells are produced through a streamlined, one‐step biological programming process, bypassing the traditional ex vivo expansion phase [50].

Transitioning from ex vivo production to in vivo generation of CAR‐T cells markedly streamlines the therapeutic workflow, reduces product heterogeneity, enables dynamic regulation, and ultimately enhances antitumor efficacy. By eliminating the need for leukapheresis, in vitro culture, large‐scale expansion, and reinfusion, in vivo CAR‐T therapy transforms treatment into a simple regimen of one or more injections [49]. This approach substantially shortens preparation timelines, lowers production costs, and improves overall accessibility [51, 52]. Moreover, in vivo CAR‐T strategies directly harness the patient's own healthy, unmanipulated T‐cell repertoire, generating more robust and functionally competent CAR‐T populations while reducing variability in the final product [53]. When non‐viral vectors delivering mRNA are employed, the short‐lived nature of mRNA‐driven CAR expression provides a built‐in regulatory “switch.” Through repeated dosing, clinicians can modulate both the intensity and duration of CAR‐T activity, providing a powerful mechanism to mitigate toxicities such as cytokine release syndrome (CRS) [54, 55]. Importantly, in vivo CAR‐T approaches may also reduce—or even eliminate—the requirement for lymphodepleting chemotherapy before treatment, offering significant improvements in safety [56, 57].

The central technological driver of in vivo CAR‐T therapy lies in the advancement of delivery vectors, which can be broadly categorized into viral and non‐viral systems [58, 59]. Viral vectors—particularly lentiviruses and adeno‐associated viruses (AAV)—enable sustained CAR expression, but their clinical use is limited by the risks of insertional mutagenesis, high immunogenicity, and elevated production costs [60, 61], prompting increased interest in non‐viral delivery platforms, which offer the potential to fundamentally optimize in vivo CAR‐T strategies. Non‐viral vectors are typified by lipid nanoparticles (LNPs) and polymer nanoparticles (PNPs) [62]. LNPs, in particular, exhibit high nucleic acid encapsulation and delivery efficiency, while offering key advantages such as non‐viral composition, low immunogenicity, scalable production, and facile chemical modification [63]. These properties make LNPs especially well‐suited for applications requiring transient gene expression and position them at the forefront of tumor immunology research [64]. Beyond oncology, the therapeutic reach of in vivo CAR‐T has been further highlighted by a landmark study from Rurik et al., who used CD5‐targeted LNPs to deliver mRNA encoding an anti‐fibrotic CAR directly into T cells. This strategy effectively generated functional CAR‐T cells in vivo and reversed cardiac fibrosis in mice, providing the initial demonstration that in vivo CAR‐T can be harnessed for non‐tumor diseases [65].

**FIGURE 1:**
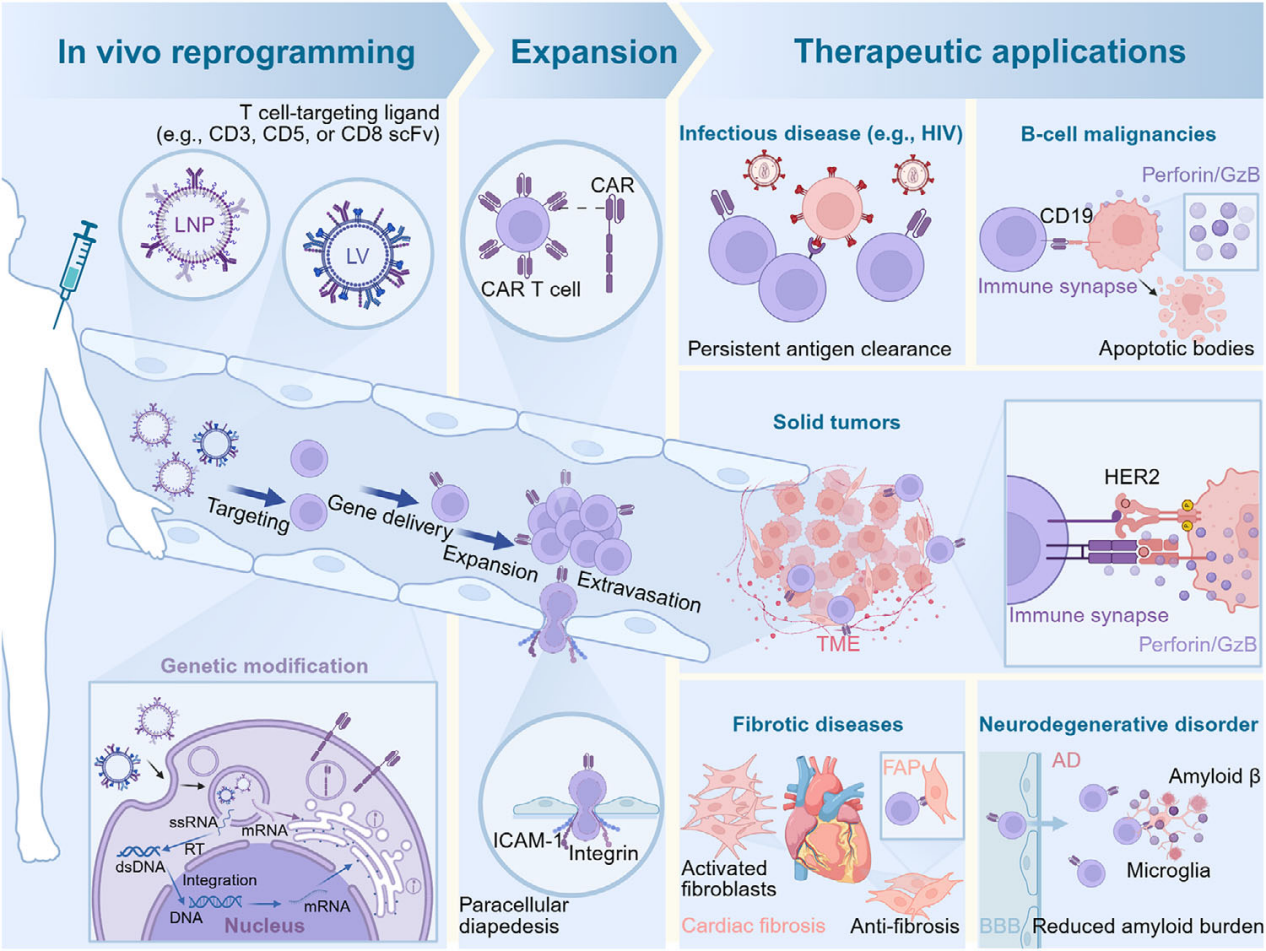
In vivo CAR‐T cell generation and indications. In vivo CAR‐T cell generation refers to the direct genetic engineering of a patient's endogenous T cells within the body. Briefly, pre‐manufactured gene delivery vectors are administered intravenously, enter the systemic circulation, and selectively target circulating T cells to deliver the CAR transgene and induce CAR expression. The engineered CAR‐T cells subsequently expand in vivo and exert antigen‐specific cytotoxicity against target‐expressing cells. Current and emerging indications for in vivo CAR‐T therapy include hematological malignancies, solid tumors, autoimmune diseases, myocardial fibrosis, and infectious diseases. Created with permission from BioRender. AD, Alzheimer's disease; BBB, blood–brain barrier; CAR, chimeric antigen receptor; dsDNA, double‐stranded DNA; FAP, fibroblast activation protein; GzB, granzyme B; HER2, human epidermal growth factor receptor 2; ICAM‐1, intercellular adhesion molecule 1; mRNA, messenger RNA; ssRNA, single‐stranded RNA; TME, tumor microenvironment.

The clinical potential of in vivo CAR‐T extends far beyond the boundaries of tumor immunology. By precisely reprogramming immune responses in vivo, this strategy has demonstrated substantial promise in autoimmune disorders [66, 67], organ fibrosis [65, 68], infectious diseases [69], and even neurodegenerative conditions [70, 71]. Collectively, these advances signal the emergence of “in vivo cell therapy” as a versatile and broadly applicable treatment platform (Figure 1). In vivo CAR‐T stands as a transformative advance in cell‐based therapies, effectively “internalizing” the traditionally complex ex vivo manufacturing workflow into the patient's own body. By doing so, it addresses the core limitations of conventional CAR‐T therapy—namely, issues related to production, accessibility, and real‐time functional regulation—thereby redefining the therapeutic landscape [72].

The objective of this review is to present a comprehensive synthesis regarding current developments and emerging research directions in in vivo CAR‐T therapy. We summarize recent advances in the field, evaluate its strengths and persistent limitations, and outline strategies for further optimization. The review is organized into four major sections, each centered on a key component of in vivo CAR‐T. Together, these sections examine recent technological advances, dissect delivery vector platforms and their unique advantages, discuss considerations for clinical translation, and provide an integrated overview of current progress while highlighting future opportunities. Through this framework, we aim to provide researchers with a broad and rigorous perspective on this rapidly evolving discipline, foster scientific innovation, and deepen our understanding of how in vivo CAR‐T may address longstanding therapeutic bottlenecks and drive meaningful advances across the medical landscape.

## 2 Chimeric Antigen Receptor T‐Cell Progress

CAR‐T therapy, a landmark innovation in tumor immunotherapy, has achieved significant clinical efficacy against malignant tumors in recent years [73]. This section provides an overview of CAR‐T's key developmental milestones, examines the intrinsic limitations of the traditional ex vivo manufacturing paradigm, and introduces the emerging in vivo CAR‐T strategy, highlighting its distinctive strengths and future potential. The conceptual foundation of CAR‐T cell therapy originated from 1989, at which time Gross et al. proposed fusing an antibody variable region with the constant region of a T‐cell receptor (TCR) to generate a synthetic antigen receptor capable of redirecting T cells to recognize and eliminate tumor cells [74, 75]. Since their inception, CAR architectures have progressed through five distinct generations [76], evolving toward increasingly modular and programmable designs. Innovations such as logic‐gated CARs, affinity‐tuned receptors, universal CAR platforms, and gene‐engineering strategies including PD‐1 knockout, suicide switches, and PROTAC‐based regulation have substantially improved the specificity, safety, and persistence of CAR‐T cells [77].

**FIGURE 2:**
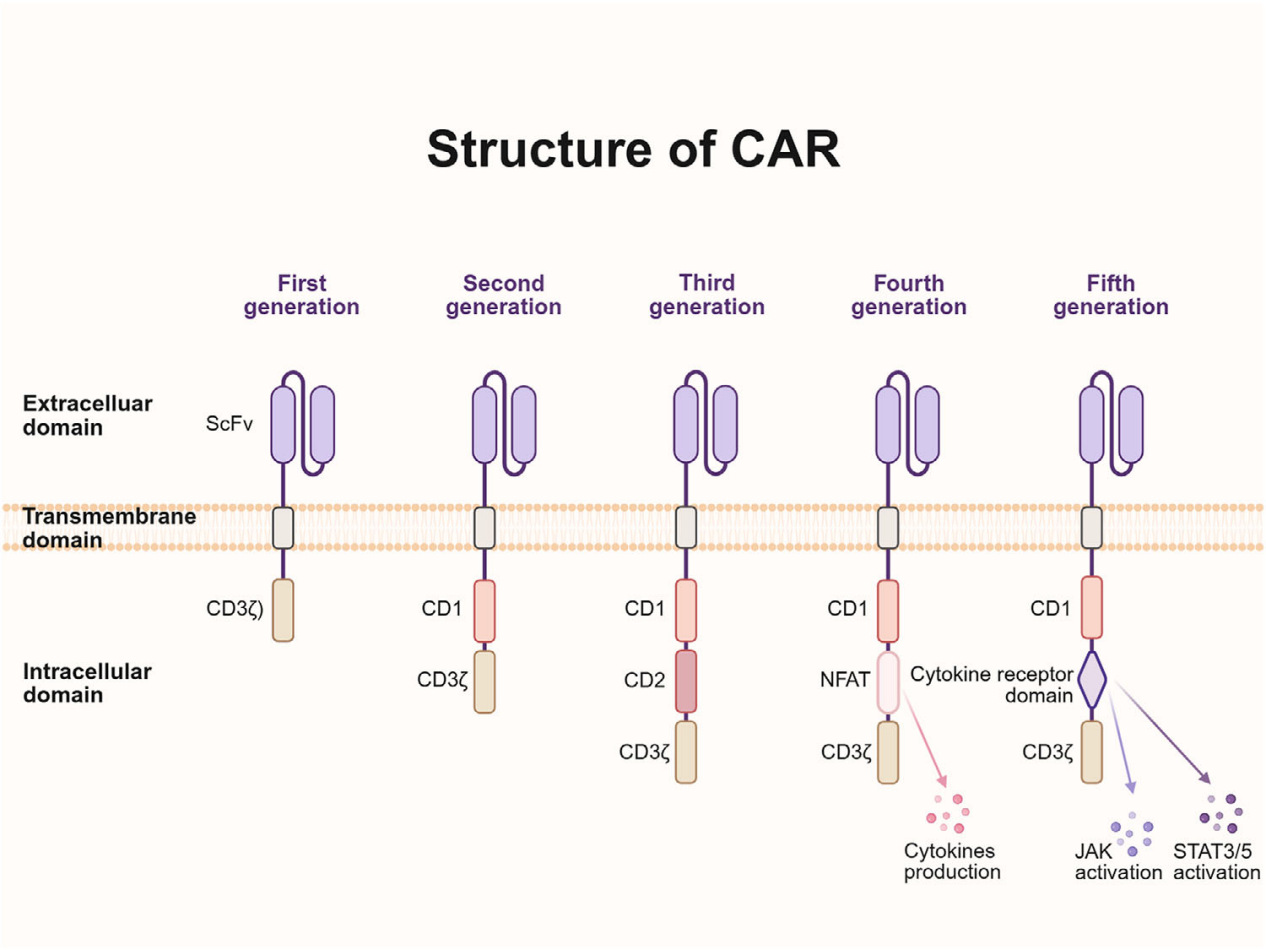
Evolution of Core CAR Technologies. The development of CAR technologies has progressed through five major generations. First‐generation CARs comprise an antigen‐binding domain (scFv), a transmembrane domain, and a CD3ζ signaling module. Second‐generation CARs incorporate a single costimulatory domain, most commonly CD28 or 4‐1BB, to enhance T‐cell activation and persistence. Third‐generation CARs tandemly combine two distinct costimulatory domains, typically CD28 and 4‐1BB, to further augment signaling. Fourth‐generation CARs build upon second‐generation backbones by integrating inducible cytokine‐expression cassettes (such as IL‐12, IL‐15, or IL‐18) or safety‐switch modules. Fifth‐generation CARs extend second‐generation constructs with cytokine receptor–derived signaling domains containing STAT3/5 transcription factor–binding motifs, enabling more precise modulation of T‐cell function. Created with permission from BioRender. CD1, costimulatory domain 1; CD2, costimulatory domain 2; JAK, Janus kinase; NFAT, nuclear factor of activated T cells; scFv, single‐chain variable fragment; STAT3/5, signal transducer and activator of transcription 3/5.

The first‐generation CAR, structurally the most basic, included an extracellular antigen‐recognition domain (scFv), a transmembrane region, and a CD3ζ intracellular signaling module [78]. Because this design provided only the “first signal” for T‐cell activation and lacked co‐stimulation, these CAR‐T cells exhibited poor expansion, limited persistence, and a high propensity for apoptosis, resulting in minimal clinical efficacy [79, 80]. To address the suboptimal activation of first‐generation constructs, researchers incorporated a co‐stimulatory domain—typically CD28 or 4‐1BB—to mimic the dual‐signal activation required for physiological T‐cell responses [81]. These second‐generation CARs significantly improved proliferation, survival, cytokine secretion, and antitumor activity [82, 83]. Based on this design, the first FDA‐approved CAR‐T products (Novartis’ Kymriah and Gilead's Yescarta) were launched in 2017 for relapsed/refractory B‐cell malignancies, achieving response rates of 80%–90% and pioneering a new phase in tumor immunotherapy [16, 23]. All CAR‐T therapies approved to date are based on second‐generation designs. Expanding on this framework, third‐generation CARs incorporate two co‐stimulatory domains (CD28 + 4‐1BB) to further potentiate activation, although their clinical superiority over second‐generation designs remains debated [84, 85]. Subsequent iterations have introduced increasing complexity and functional specialization: fourth‐generation CARs (TRUCKs) incorporate inducible cytokine expression to remodel the TME and recruit innate immune pathways [86, 87], while fifth‐generation CARs embed cytokine receptor signaling motifs to generate triple‐signal activation (Figure 2), aiming to address the challenges posed by the immunosuppressive microenvironment of solid tumors [84, 88]. Nevertheless, most in vivo applications remain at the preclinical stage.

Despite the remarkable clinical efficacy of second‐generation CAR‐T therapy in hematologic malignancies [89] and emerging advances in overcoming the immunosuppressive TME of solid tumors [90], progress has been achieved through several engineering strategies. For instance, T cells Redirected for Universal Cytokine‐mediated Killing (TRUCKs) are designed to secrete pro‐inflammatory cytokines, thereby remodeling the TME and converting immunologically “cold” tumors into “hot” tumors [53]. Beyond cytokine “armoring,” another advanced in situ engineering strategy involves the use of gene‐editing tools to disrupt immune checkpoint genes during CAR‐T cell generation, thereby effectively blocking PD‐L1‐mediated immunosuppressive signaling within the TME [91, 92, 93]. Collectively, these approaches endow CAR‐T cells with the capacity to actively modulate the TME, enabling their antitumor activity in otherwise highly immunosuppressive solid tumor settings. However, with increasing clinical application, the limitations of the ex vivo manufacturing paradigm have become more evident, substantially constraining further development [17].

These limitations are concentrated in three interrelated technical bottlenecks: manufacturing complexity, cellular product quality, and dynamic control. Conventional CAR‐T therapy relies on a highly individualized workflow for producing a “living drug,” involving leukapheresis, T‐cell isolation, activation, CAR gene transduction, ex vivo expansion, quality testing, and reinfusion [94]. This complex procedure typically takes 3–5 weeks and incurs substantial costs, often amounting to hundreds of thousands of US dollars per treatment [95, 96]. The procedure further depends on an extensive global cold‐chain infrastructure, and for patients with rapidly progressing disease, such prolonged manufacturing timelines can be fatal [17]. Compounding these logistical challenges is the variability of the final product. Patients with advanced malignancies who have undergone multiple rounds of cytotoxic therapy frequently exhibit compromised T‐cell quantity and functionality, making it difficult to generate sufficient numbers of potent CAR‐T cells ex vivo. Moreover, prolonged in vitro expansion often drives T cells toward an exhausted phenotype, diminishing their proliferative capacity and functional durability after reinfusion [97]. The absence of precise dynamic regulation of CAR‐T cell activity further limits long‐term therapeutic efficacy [98, 99]. These accumulating obstacles have slowed the progression of ex vivo CAR‐T approaches. In contrast, the emergence of in vivo CAR‐T provides a compelling solution.

**FIGURE 3:**
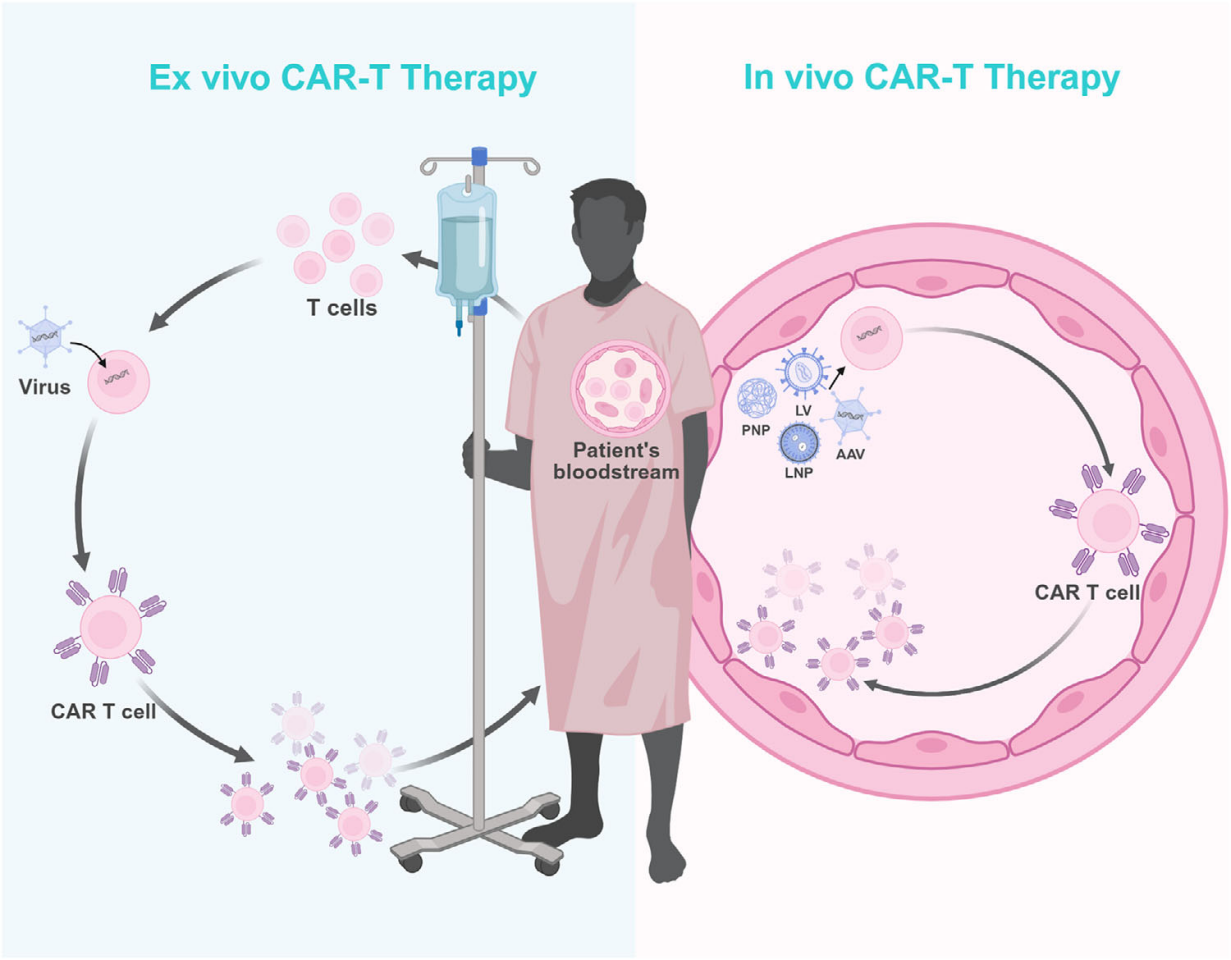
Comparison of ex vivo versus in vivo CAR‐T Treatment Workflows. CAR‐T therapy can be implemented via ex vivo or in vivo approaches, each with distinct advantages. In the ex vivo workflow, T cells are isolated from the patient through leukapheresis and genetically modified using viral or non‐viral vectors. These engineered cells are expanded to clinically relevant numbers before the patient receives lymphodepleting chemotherapy and subsequent infusion. In contrast, in vivo CAR‐T therapy employs “off‐the‐shelf” gene‐delivery vectors administered intravenously. These vectors selectively transduce T cells in situ, generating CAR‐T cells within the patient, which then undergo activation and expansion to directly target tumor cells. Created with permission from BioRender. AAV, adeno‐associated virus; LNP, lipid nanoparticle; LV, lentiviral vector; PNP, polymer nanoparticle.

By relocating the CAR‐T “manufacturing factory” from external facilities to the patient's own body, this strategy enables in situ and on‐demand T‐cell reprogramming through one or several injections, thereby addressing many of the foundational limitations of the ex vivo model (Figure 3). In vivo CAR‐T redefines the therapeutic paradigm by shifting from cellular products to gene‐based medicines [13]. This approach employs specialized delivery vectors—engineered viral platforms or nanoparticles—to selectively target T cells and deliver CAR‐encoding DNA or mRNA directly in vivo, thereby enabling endogenous T cells to express CAR molecules and convert into functional CAR‐T cells within the patient [100, 101]. Compared with conventional ex vivo therapy, this strategy offers several compelling advantages: streamlined manufacturing, reduced costs, improved cellular quality, and controllable, repeatable administration. By enabling the immediate administration of “off‐the‐shelf” gene drugs, in vivo CAR‐T obviates the laborious ex vivo preparation steps, which is expected to markedly reduce production expenses, lower treatment thresholds, and improve accessibility [102]. Moreover, this strategy leverages the patient's native, younger, unmanipulated T cells, which have not undergone the stresses of in vitro activation and expansion, thereby generating CAR‐T cells with improved functional capacity and diminished exhaustion [99]. Because administration occurs ex vivo while CAR‐T activity is induced in vivo, treatment schedules can be flexibly modulated to shape the activation window of CAR‐T cells [103], improving the capacity to mitigate toxicities such as CRS and enabling more effective control of relapse [55, 104].

In vivo CAR‐T strategies can not only generate highly functional CAR‐T cells with reduced exhaustion but may also confer phenotypic and therapeutic characteristics distinct from those of conventional CAR‐T cells. In the MASTER implantable scaffold study, in vivo‐generated CAR‐T cells contained higher proportions of central memory T cells (14.8% vs. 2.57%), stem‐like T cells (21.5% vs. 9.8%), and T cells with lymphoid homing capacity (13.1% vs. 2.98%) than conventionally manufactured CAR‐T cells [52]. Unlike conventional ex vivo CAR‐T manufacturing, in vivo programming occurs within the patient's endogenous immune system and may therefore preserve T‐cell subsets that are progressively lost or differentiated during prolonged ex vivo culture. This feature may be particularly advantageous for generating CAR‐T cells with stem‐like and memory phenotypes, which could support sustained proliferative capacity and antitumor activity following antigen stimulation. Thus, in vivo CAR‐T not only simplifies the manufacturing process but may also reshape the composition, cellular fitness, and functional state of therapeutic T cells [57].

Building on these advantages, the therapeutic scope of in vivo CAR‐T has gradually expanded beyond conventional oncology indications. In 2023, Billingsley et al. developed CD3‐ and CD7‐targeted ionizable LNPs (Ab‐LNPs) for the delivery of CD19 CAR‐encoding mRNA, which generated functional CAR‐T cells in mice and induced substantial B‐cell depletion [103]. In 2024, the VivoVec platform generated anti‐CD20 CAR‐T cells accounting for up to 65% of circulating T cells in non‐human primates (NHPs) (cynomolgus monkeys), resulting in complete B‐cell depletion for up to 76 days [57]. Notably, B‐cell repopulation following depletion was predominantly characterized by naive B cells, suggesting that this approach may have the potential to induce an “immune reset.” Such an ability is particularly relevant to the treatment of autoimmune diseases, in which depletion of pathogenic B cells followed by reconstitution of the B‐cell compartment may provide an opportunity to restore immune tolerance.

In vivo CAR‐T strategies for autoimmune diseases have attracted increasing attention and have now entered clinical evaluation. In 2025, a clinical study reported the in vivo generation of CD19 CAR‐T cells using LNPs in patients with refractory systemic lupus erythematosus, resulting in B‐cell depletion and reduced disease activity without significant severe toxicities [105]. In addition, an ongoing Phase 1 clinical trial (NCT07413341) is evaluating an LNP‐based platform for in vivo generation of CD19 CAR‐T cells using circular RNA in patients with relapsed or refractory B‐cell‐mediated autoimmune diseases, including systemic lupus erythematosus, Sjogren's syndrome and systemic sclerosis. These advances indicate that, beyond cancer immunotherapy, autoimmune diseases may represent a particularly promising setting for the early clinical translation of in vivo CAR‐T strategies.

Translating these advances in oncology and autoimmune diseases into broadly applicable therapeutic modalities will require a shift from proof‐of‐concept efficacy toward predictable in vivo cell manufacturing. In conventional CAR‐T therapy, the cellular product can be extensively characterized before infusion, whereas the number, phenotype, and functional state of in vivo‐generated CAR‐T cells cannot be directly characterized to the same extent and may vary according to endogenous T‐cell abundance, immune status, and disease context [53, 106]. Future development will therefore require precise monitoring and control of in vivo transduction specificity, CAR expression, cellular expansion and persistence [107]. Establishing such control frameworks will be critical for achieving reproducible therapeutic responses and predictable safety profiles across patients, thereby facilitating the clinical translation of in vivo CAR‐T.

Beyond CAR‐T cells, in vivo CAR‐NK cell engineering has attracted increasing attention. This approach enables the generation of engineered NK cells directly in patients via CAR gene delivery, bypassing ex vivo manufacturing constraints [108, 109, 110]. Key design considerations include targeting specificity, expression duration, and safety. Targeting strategies often focus on NK‐associated surface markers such as CD7, as exemplified by INT2104, a CD7‐targeted lentiviral vector (LV) selectively transducing CD7^+^ T and NK cells [48]. Viral vectors such as lentiviruses enable durable expression but carry risks of insertional mutagenesis, whereas mRNA–LNPs provide transient expression with improved safety, albeit with a short expression window (approximately 4–5 days), potentially requiring repeated dosing or self‐amplifying RNA approaches [111, 112]. Compared with CAR‐T cells, CAR‐NK cells exhibit lower risks of CRS, neurotoxicity, and graft‐versus‐host disease, and they mediate MHC‐independent cytotoxicity via both CAR‐dependent and innate receptors such as NKG2D and NKp30 [109]. However, major challenges remain, including low viral transduction efficiency, short in vivo persistence, and sensitivity to the immunosuppressive TME, which may necessitate cytokine support (e.g., IL‐15) or metabolic reprogramming to enhance function [111, 113, 114]. Additional safety concerns include immune responses to delivery vectors and potential toxicities associated with in vivo‐generated CAR immune cells [53].

Although in vivo CAR‐T and CAR‐NK therapies hold transformative promise for simplifying manufacturing, improving cellular fitness, and enabling greater therapeutic control, significant challenges remain before these technologies can be successfully translated from conceptual advances into clinical reality. Key hurdles include effective and selective in vivo targeting of T cells, avoidance of off‐target effects, and ensuring predictable and durable CAR expression. Despite these challenges, several in vivo CAR‐T approaches have already entered clinical trials, marking an important step toward clinical translation (Table 1). Further optimization is nevertheless required to enhance safety and efficacy, facilitate the transition from preclinical development to scalable manufacturing, and enable robust and controllable in vivo CAR‐T cell generation. Overall, as a pioneering technological innovation, in vivo CAR‐T substantially refines conventional CAR‐T paradigms and holds the promise of converting CAR‐T therapy from an expensive, highly customized intervention into a broadly accessible, standardized treatment modality. The following section will examine the major classes of in vivo CAR‐T delivery vectors and provide a systematic analysis of their respective advantages.

**TABLE 1:** Advancements in in vivo CAR‐T clinical trials.

Vector	Product	Institution	CAR target	Indications	Phase	NCT number
LV	ESO‐T01	EsoBiotec	BCMA	B‐cell tumors	Phase 1	NCT06691685
	JY‐231	Shenzhen Genocury Biotech	CD19	B‐cell tumors	Phase 1	NCT06890065
	UB‐VV111	Umoja Biopharma	CD19	B‐cell tumors	Phase 1	NCT06528301
	LVIVO‐TaVec100	Legend Biotech	CD19/CD20	B‐cell tumors	Phase 1	NCT07002112
	INT2104	Interius BioTherapeutics	CD20	B‐cell tumors	Phase 1	NCT06539338
	UB‐V400/410	Nanjing IASO Biotechnology	CD22	B‐cell tumors	Phase 1	NCT06743503
	KL‐1010	CSPC ZhongQi Pharmaceutical	BCMA	B‐cell tumors	Phase 1	NCT06688435
LNP	JCXH‐213	Immorna Biotherapeutics	CD19	B‐cell tumors	Phase 1	NCT06618313
	CPTX2309	Capstan Therapeutics	CD19	Autoimmune diseases	Phase 1	NCT06917742
	HN2301	Shenzhen MagicRNA	CD19	Autoimmune diseases	Phase 1	NCT06965309

*Note*: Data sources—ClinicalTrials.gov.

## 3 In Vivo Chimeric Antigen Receptor T‐Cell Delivery System

**FIGURE 4:**
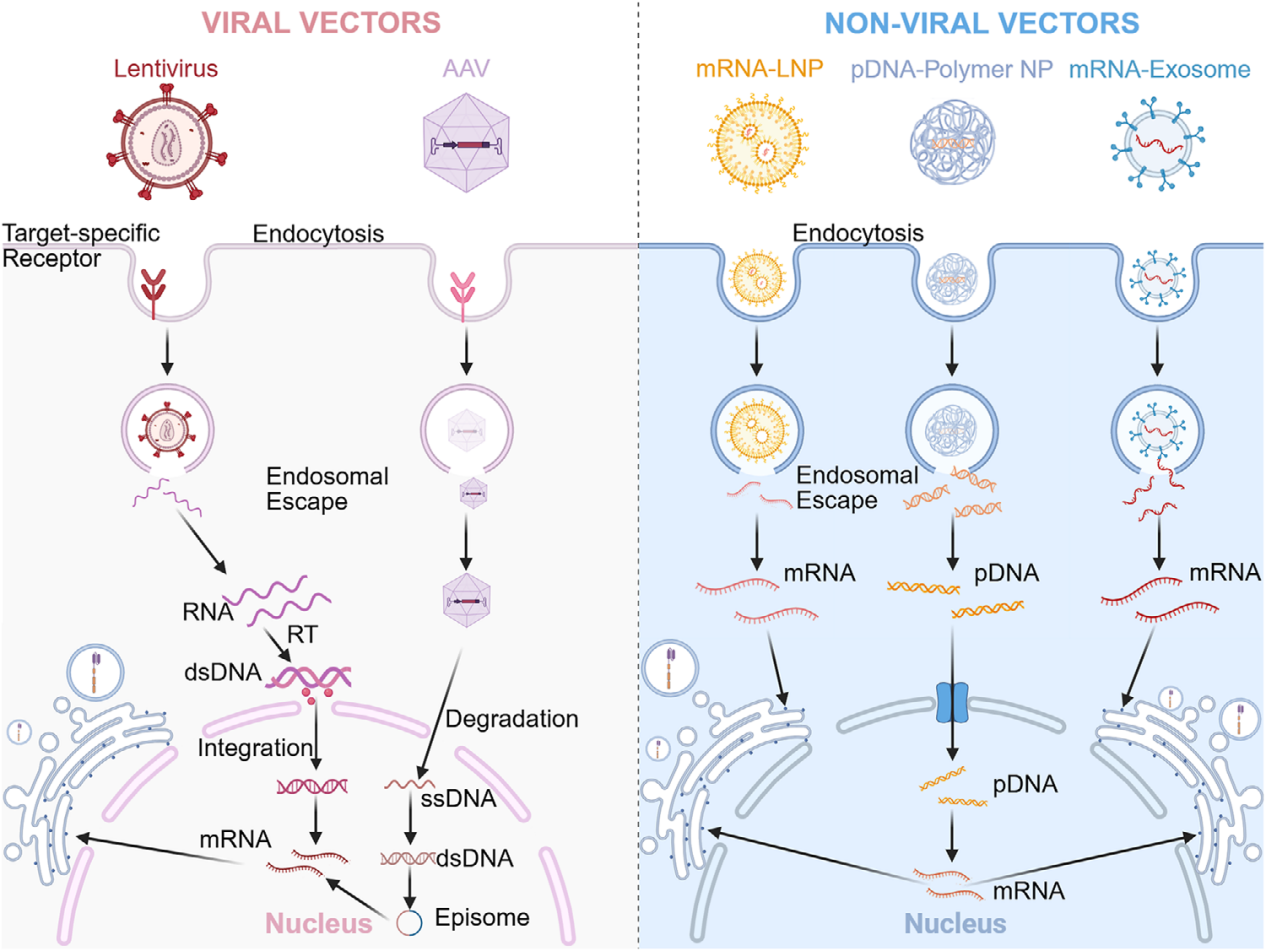
Multiple vectors for in vivo CAR‐T cell generation. Delivery vehicles for in vivo CAR‐T generation can be broadly classified into viral and non‐viral vectors. Viral vectors are primarily represented by lentiviruses and adeno‐associated viruses, whereas non‐viral vectors include lipid nanoparticles (LNPs), polymer nanoparticles, and exosomes. These carriers can deliver nucleic acid cargoes such as DNA or RNA, typically entering T cells via endocytosis. Following intracellular trafficking, cargo release, and gene expression, CAR proteins are ultimately produced and localized to the cell membrane. Created with permission from BioRender. AAV, adeno‐associated virus; LNP, lipid nanoparticle; NP, nanoparticle; RT, reverse transcription.

In vivo CAR‐T therapy operates by administering a delivery system that carries the CAR‐encoding genetic material. Once introduced into the body, this system targets T cells—or, in some cases, other immune cell populations—to enable in situ genetic engineering and subsequent CAR expression [48]. As the central mediator of this process, the delivery system is pivotal: its design and performance determine whether CAR DNA or mRNA can be delivered to target immune cells safely, efficiently, and with high specificity. At present, two major classes of vectors dominate the field: viral vectors and non‐viral vectors [59]. In addition to these conventional platforms, emerging nanoparticle‐based injection technologies have also attracted considerable attention. Viral and non‐viral vectors follow distinct technological trajectories and exhibit complementary advantages (Table S1), together driving the rapid development of in vivo CAR‐T platforms (Figure 4). The continued refinement of these delivery systems is expected to advance the future development of in vivo CAR‐T therapy.

### 3.1 Viral Delivery System

Over millions of years of evolutionary pressure, viruses have evolved potent systems for both cell entry and genetic cargo delivery. This intrinsic capability has made viral vectors a cornerstone in gene therapy [115]. For in vivo CAR‐T applications, lentiviruses and AAVs are the two best‐characterized viral delivery systems, each offering distinct advantages. Lentiviruses are particularly noteworthy for their ability to stably integrate the CAR gene into the genome of host T cells [116], thereby potentially endowing CAR‐T cells with long‐lasting functionality. Although the VivoVec platform achieved complete B‐cell depletion for up to 76 days in NHPs [57], persistent antigen stimulation may increase the risk of T‐cell exhaustion [53]. Notably, engineered VivoVec‐derived CAR‐T cells exhibited higher expression of CCR7 and CD27, suggesting an improved memory phenotype and enhanced persistence potential [57]. In contrast, AAVs typically exist as episomal elements, allowing for transient expression [117]. Despite this, AAVs are favored for their lower immunogenicity and superior in vivo delivery efficiency [118], making them an ideal choice for generating rapid, short‐term CAR‐T responses with potent antitumor effects.

#### 3.1.1 Lentiviral Vector

LVs represent highly promising tools in gene delivery, capable of accommodating large exogenous gene constructs [119], thereby facilitating the incorporation of multifunctional CAR designs. Their ability to infect non‐dividing cells, such as T lymphocytes, and to stably integrate exogenous genes into the host genome makes LVs particularly advantageous for generating long‐term [120], persistent CAR‐T cell populations. In addition, LVs exhibit exceptional transduction efficiency in hematopoietic cells [121], including T cells, ensuring robust and durable gene expression.

To optimize the specificity and safety of LVs, researchers have employed genetic engineering strategies to alter their tropism and reduce the risk of insertional mutations. While natural LVs possess broad‐spectrum host cell tropism, leading to non‐specific transduction in vivo and an associated potential for unintended genomic integration, targeted modifications can mitigate this issue [122]. For example, envelope pseudotyping—which involves fusing anti‐CD3, CD4, or CD8 scFvs to the viral envelope, or displaying other targeting motifs like ankyrin repeat proteins or camelid single‐domain antibodies—can direct the virus to specific T cell subsets [123, 124, 125]. Notable advancements include the work of Krug et al., who developed an LV targeting CD8 and successfully generated CAR‐T cells that specifically targeted angioimmunoblastic T‐cell lymphoma in vivo [126]. Similarly, Frank et al. created an LV targeting CD3, enabling direct infection and activation of quiescent T cells without the need for pre‐activation, thereby simplifying the in vivo CAR‐T generation process [127]. To additionally mitigate the risk of insertional mutagenesis, current LVs are often engineered with a “self‐inactivation” design. This modification, which deletes enhancer or promoter sequences in the viral long terminal repeat (LTR) region, significantly reduces the likelihood of activating proto‐oncogenes through random integration [119]. As of now, LV‐based in vivo CAR‐T therapies have entered Phase I clinical trials, signaling an exciting step toward their broader application in tumor immunotherapy.

LV‐based in vivo CAR‐T therapies are currently in Phase I clinical trials, but progress is advancing rapidly. The most common targeting strategies under investigation include CD3, CD7, and multi‐domain approaches, with CD3‐targeting remaining the primary focus. Notably, the ESO‐T01 trial demonstrated the feasibility of this strategy, achieving a 100% objective response among individuals with relapsed and refractory multiple myeloma, providing strong evidence for the therapeutic potential of LV‐based in vivo CAR‐T therapy [128]. The CD7‐targeting strategy represents a more novel approach, as it not only facilitates the transduction of T cells but also enables the targeting of natural killer (NK) cells, thereby expanding the effector cell lineage and potentially enhancing therapeutic efficacy (NCT06539338). In parallel, multi‐domain and multi‐target strategies are pioneering advancements in CAR‐T engineering, designed to more closely mimic natural immune activation in vivo. These strategies seek to improve the quality of CAR‐T cells by generating stronger, less exhausted cells that are more resilient and functional in the TME (NCT06743503).

#### 3.1.2 Adeno‐Associated Virus Vector

In addition to LVs, AAVs present significant merits as gene delivery systems, with features like minimal immunogenicity and non‐integrative properties—the latter allowing AAVs to form stable episomes rather than integrating into the host genome [129]. These characteristics make AAVs a widely utilized tool in gene therapy [130]. AAVs consist of several serotypes, each with distinct tissue and cell tropisms, enabling targeted delivery to specific immune cells [131]. Furthermore, their non‐integrative nature minimizes the risks of genotoxicity and oncogenesis. In non‐dividing cells, AAV episomes can persist stably, providing the potential for long‐term gene expression, lasting for months or even longer.

However, despite their natural advantages, AAVs have certain limitations when compared to LVs. Although the limited packaging capacity of AAV (∼4.7 kb) has long been considered a major constraint for accommodating complex CAR constructs and additional regulatory elements [132], many modern CAR designs, particularly those incorporating compact scFvs, can now be efficiently packaged within AAV vectors. In addition, due to prior natural infections, many individuals harbor neutralizing antibodies against specific AAV serotypes, which can hinder the therapeutic effectiveness and contribute to possible treatment failure [133]. Another key limitation is the lack of inherent tropism for T cells, which results in a substantially lower transduction efficiency compared to LVs. This limitation poses a major obstacle to the widespread use of AAV in in vivo CAR‐T therapies [134]. Although several studies have demonstrated the feasibility of AAV‐mediated intravenous delivery of CAR transgenes for the treatment of T‐cell leukemia [60], recent advances have identified an engineered AAV6 variant, AAV6‐M2, that exhibits enhanced transduction efficiency in human T cells through CD62L‐mediated targeting and enables the generation of functional CAR‐T cells with a favorable safety profile [135]. Nevertheless, the broader applicability and therapeutic efficacy of AAV‐based in vivo CAR‐T cell engineering warrant further investigation and optimization.

### 3.2 Non‐Viral Delivery System

While viral vectors offer distinct advantages in gene delivery, their inherent immunogenicity introduces potential safety concerns. In contrast, non‐viral vectors have attracted considerable interest due to their minimal immunogenicity, enhanced safety profiles, and ease of production. Key non‐viral vectors, for example, LNPs and PNPs, are particularly promising owing to their potential to address several limitations associated with viral systems [63]. The primary advantages of non‐viral vectors include their low immunogenicity and high safety, along with standardization and large‐scale production capabilities. These factors contribute to significantly reduced costs and shorter preparation timelines, making them highly attractive for clinical applications [136]. Furthermore, non‐viral systems enable flexible dosing and transient CAR expression, allowing precise control over CAR‐T cell activity. Because CAR expression is generally transient and lasts only a few days [55], this strategy may mitigate the risk of T‐cell exhaustion driven by persistent antigen stimulation [137]. Moreover, the avoidance of ex vivo culture‐induced differentiation may favor the generation of T cells with enhanced stem‐like and memory characteristics [50]. These properties can be further reinforced through the co‐delivery of metabolism‐enhancing mRNAs, such as TERT, which have been shown to improve cellular fitness and proliferative potential [138]. This flexibility not only strengthens the safety aspects of the therapy but also allows for dynamic modulation of the CAR‐T response, optimizing therapeutic outcomes.

#### 3.2.1 Lipid Nanoparticle Carrier

LNPs have arisen as top‐tier carriers for non‐viral gene delivery [139]. Their structure is composed of a self‐assembled core made from a combination of ionizable lipids, helper phospholipids, cholesterol, and PEGylated lipids, which collectively enable the efficient encapsulation of nucleic acids—both RNA and DNA—while protecting them from degradation by nucleases [140, 141]. The efficiency of LNP‐mediated delivery is largely dictated by the properties of ionizable lipids, which undergo protonation in acidic environments. This protonation enhances fusion with cell membranes, promoting the release of genetic cargo into the cytoplasm for subsequent expression [142].

In in vivo CAR‐T strategies, LNPs primarily serve as vehicles for delivering either mRNA or DNA. Compared to DNA, mRNA is smaller, functions directly in the cytoplasm, and offers several distinct advantages, including high safety, transient expression, and the ability to avoid genomic integration [140]. This makes mRNA‐based delivery one of the most mature and advanced technologies currently available. LNP‐delivered mRNA undergoes translation into protein in the cytoplasm, bypassing the nucleus and thereby fundamentally reducing the risk of genomic integration‐related mutations [143]. In addition, the transient nature of mRNA expression acts as an intrinsic “safety switch,” which can be leveraged to finely tune CAR‐T cell activity and duration of effect. This feature allows for repeated administrations and better management of side effects, including CRS [55, 144]. Lemgart et al. developed CD8‐targeted nanobody‐conjugated LNPs that efficiently generated functional CAR‐T cells in vivo, resulting in substantial reductions in tumor burden while allowing repeated dosing without significant toxicity [145]. Beyond CAR transgene delivery, LNPs have been adapted for multiplexed RNA delivery, enabling the co‐delivery of CAR‐ and Cas9‐encoding mRNAs together with sgRNAs targeting PD‐1, TRAC, and B2M [146]. This approach supports simultaneous CAR expression and genome editing, thereby improving engineering efficiency while preserving T‐cell viability and facilitating the generation of multiplex‐engineered CAR‐T cell products. While mRNA is the predominant cargo, LNPs also have the capacity to deliver DNA. However, DNA molecules are larger and require nuclear entry to integrate into the host genome, presenting a greater challenge for delivery. Despite this, recent work has illustrated the potential of LNP‐based DNA delivery. For instance, Bimbo et al. utilized LNPs to deliver mcDNA along with SB100x transposase mRNA, achieving stable integration of the CAR gene sequence into the T cell genome. This approach resulted in long‐term CAR expression, and a single injection was sufficient to cure a mouse model of leukemia [47].

The use of LNP vectors for efficient nucleic acid delivery offers substantial advantages, and research in tumor immunity leveraging this platform is progressing rapidly [147]. The successful development of LNP‐based mRNA vaccines, such as those for COVID‐19, has provided a critical technological foundation for their application in the CAR‐T field [139]. LNPs can also facilitate the transfer of CRISPR/Cas9 gene editing components, supporting accurate gene knockouts or targeted insertion of CAR genes into T cells in vivo [148]. Building on this progress, numerous companies are now focusing on the clinical translation of LNP‐mRNA technologies. These efforts can be broadly categorized into two strategies: cell‐targeting LNPs and cell‐loving LNPs. Currently, LNP‐DNA delivery remains primarily in the preclinical stage but is a promising direction for future development. T cell‐targeting LNPs are engineered with specific antibodies conjugated to their surface, enhancing delivery precision. For instance, CPTX2309 uses CD8‐targeting LNPs to encapsulate mRNA encoding an anti‐CD19 CAR and has already entered Phase 1 clinical trials [149]. Other companies, such as Shenzhen MagicRNA and Immorna, are adopting similar antibody‐targeting strategies, with a focus on CD19, and are targeting tumors and autoimmune diseases. Even more innovative are myeloid cell tropism‐type LNPs, which specifically target myeloid cells and are primarily aimed at solid tumors. Myeloid Therapeutics has developed LNP‐CAR therapies targeting TROP2 (MT‐302) and GPC3 (MT‐303), both currently in Phase I clinical trials (NCT05969041, NCT06478693) for the treatment of epithelial malignancies and hepatocellular carcinoma.

#### 3.2.2 Polymer Nanoparticles and Other Delivery Systems

In addition to LNPs, other non‐viral vectors such as PNPs and innovative vectors are gaining attention for in vivo CAR‐T applications. PNPs represent a crucial class of gene delivery systems, utilizing cationic polymers to form stable complexes with negatively charged nucleic acids through electrostatic interactions [150]. These vectors are highly modifiable, allowing them to carry a variety of therapeutic payloads; however, their biocompatibility remains an area for improvement. Other emerging delivery systems, such as exosomes and implantable scaffolds [151, 152], have attracted considerable attention because of their superior biocompatibility and potential for targeted delivery.

PNP vectors are primarily based on cationic polymers, including poly(β‐amino ester) (PBAE) and polyethyleneimine (PEI) [153]. The chemical structures of these polymers can be precisely engineered to optimize delivery efficiency, degradation rates, and biocompatibility [154]. Cationic polymers, when used to encapsulate nucleic acids, exhibit a “proton sponge” effect, which facilitates endosomal escape and enhances the efficiency of nucleic acid delivery [150]. Furthermore, PNPs can carry multiple therapeutic agents simultaneously, such as CAR genes and immunomodulatory factors, enabling synergistic therapies. PNPs have demonstrated their potential in CAR‐T therapies. For example, Smith et al. successfully coupled PBAE nanoparticles with the F(ab’)_2_ fragment of an anti‐CD3 antibody to deliver DNA plasmids encoding CD19‐CAR, achieving in vivo T cell reprogramming in a mouse leukemia model [50]. In addition, Moffett et al. used PBAE polymers to deliver CAR‐mRNA, further demonstrating the suitability of PNPs for therapeutic applications requiring controllable expression of the CAR construct [155].

PNP vectors offer advantages in terms of ease of production and regulation; however, many highly efficient PNPs, such as PEI, exhibit cytotoxicity due to their high positive charge density, which compromises their biocompatibility [63]. As a result, current research predominantly utilizes PBAE, which demonstrates lower toxicity [153]. Jain et al. developed biodegradable PBAE‐based mRNA nanoparticles functionalized with dual ligands targeting CD3 and CD28. This design enabled the simultaneous targeting, activation, and transfection of T cells, thereby providing an integrated platform for efficient in vivo T‐cell engineering [156]. In addition to PNPs, other innovative vectors have gained significant attention for their high biocompatibility, including exosomes and implantable scaffolds. Exosomes, as endogenous natural nanomaterials, offer excellent biocompatibility and low immunogenicity, making them highly promising as gene delivery vehicles [157]. Implantable scaffold systems represent another innovative approach that aims at overcome the barriers posed by solid tumors. These systems involve implanting biomaterials carrying CAR constructs directly into the tumor, where they recruit and reprogram T cells in situ, facilitating local and efficient CAR‐T cell generation [52]. Non‐viral vectors provide several key benefits, including higher safety, ease of large‐scale production, and the ability to implement flexible and controllable treatment regimens, positioning them as an important avenue for the clinical translation of in vivo CAR‐T.

Beyond conventional non‐viral vectors, emerging delivery strategies have gained increasing attention. Over the past decade, nanostructure‐mediated intracellular delivery systems have advanced substantially, culminating in the development of dedicated nanoinjection platforms for cell engineering applications [158]. These technologies have demonstrated efficient delivery of nucleic acids, mRNA, proteins, and gene‐editing cargo while maintaining high cell viability and functionality [159]. They also exhibit excellent biocompatibility and low safety risks across diverse cell types [160], with the potential to substantially reduce manufacturing costs and improve accessibility.

Nanoinjection technologies have already been applied in immune cell engineering and CAR cell manufacturing [161]. Representative examples include silicon nanotube‐mediated non‐viral CAR‐T generation [162], electroactive nanoinjection for CAR‐T engineering [163], and nanoneedle‐based electroporation for producing primary human CAR‐regulated T cells [164]. These approaches enable the generation of CAR‐T products with improved functionality and higher purity, while indirectly addressing key limitations of conventional viral delivery, including immune clearance and biosafety concerns. Although nanoinjection platforms are currently mainly used for ex vivo cell engineering, they also provide innovative opportunities for in vivo CAR‐T strategies. Recent reviews have positioned nanoinjection as an emerging platform for ex vivo cell engineering, advanced biomanufacturing, mechanobiology‐guided cellular programming, genome editing, and next‐generation immunotherapies [165, 166]. In the previous section, we categorized and introduced various delivery vectors for in vivo CAR‐T, with each presenting a distinct set of benefits. However, the success of these delivery systems in clinical applications will depend on their ability to function safely and efficiently in the complex in vivo environment [167]. In the following section, we will categorize and summarize the existing limitations of in vivo CAR‐T and propose corresponding optimization strategies, seeking to offer deeper insights into the clinical translation of this innovative therapy.

## 4 Clinical Translation Strategy

**FIGURE 5:**
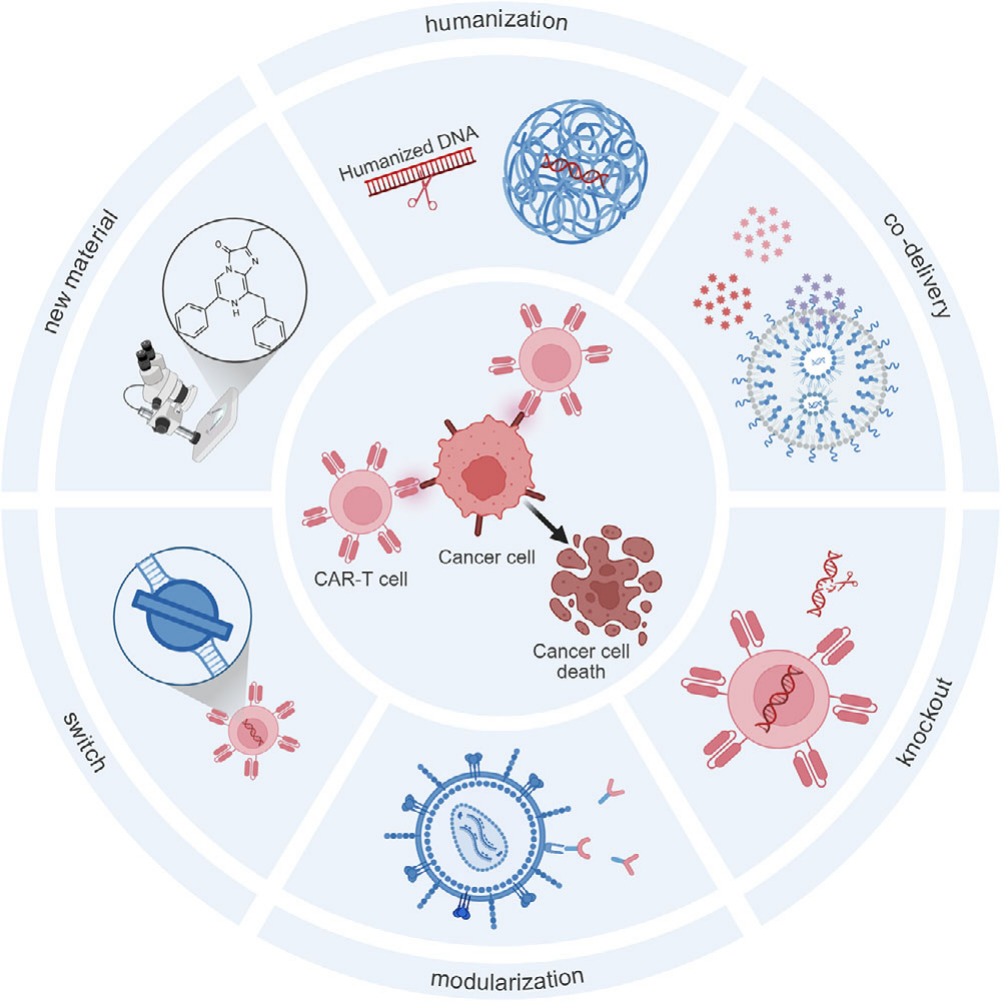
Optimization strategies for the clinical application of in vivo CAR‐T therapy. Clinical translation of in vivo CAR‐T therapy relies on multiple complementary strategies. These include the development of novel biomaterials, modular delivery vectors, and humanized CAR sequences or gene‐editing platforms to optimize construct design. Additional approaches focus on functional control, such as incorporating molecular switches, introducing suicide genes, or employing co‐delivery systems to remodel the tumor microenvironment. Furthermore, CRISPR‐mediated ablation of inhibitory receptors on CAR‐T cells can mitigate T‐cell exhaustion and enhance antitumor efficacy. Created with permission from BioRender.

While the vectors discussed above each offer distinct advantages and provide a solid technological foundation for in vivo CAR‐T development, the clinical translation of these new technologies is a complex, multifaceted systems engineering challenge. Shifting from the idealized laboratory environment to the dynamic and heterogeneous human in vivo system reveals a host of challenges that may have been underappreciated or overlooked in in vitro experiments. The following section will focus on analyzing optimization strategies for in vivo CAR‐T in terms of safety and efficacy (Table 2), with the goal of facilitating the advancement of clinical translation (Figure 5).

**TABLE 2:** Efficacy and safety profiles of major vector platforms in preclinical studies.

Delivery platform	Delivery system	CAR target	Indications	Efficacy	Safety	References
LV	CD4‐LV	CD19	B‐cell tumors (ALL)	Complete response	1. T cell exhaustion 2. B‐cell exhaustion	[123]
	CD8‐LV	CD19	B‐cell tumors (ALL)	Complete response	1. T cell exhaustion 2. B‐cell exhaustion	[225]
	CD8‐LV	CD19	B‐cell tumors (BL)	Partial response	1. CRS symptoms 2. Neurotoxicity 3. B‐cell aplasia	[53]
	CD8‐LV	CD4	T‐cell tumors (AITL)	Partial response	1. T cell exhaustion	[126]
	VivoVec	CD20	B‐cell tumors (ALL)	Complete response	1. B cell exhaustion	[57]
	VivoVec	CD19	B‐cell tumors (ALL R/R DLBCL)	Complete response	Not reported	[56]
AAV	AAV‐CD4CAR	CD4	T‐cell tumors (ATL)	Partial response	1. T cell exhaustion	[60]
LNP	NCtx	CD19	B‐cell tumors (ALL)	Complete Response	1. T cell exhaustion 2. B cell exhaustion	[47]
	t‐LNPs	FAP	Steatohepatitis (MASH)	Partial response	Not reported	[137]
Implantable stent	MASTER	CD19	B‐cell tumors (Burkitt)	Partial response	Not reported	[52]

Abbreviations: AITL, angioimmunoblastic T‐cell lymphoma; ALL, acute lymphoblastic leukemia; ATL, adult T‐cell leukemia; BL, Burkitt lymphoma; MASH, metabolic dysfunction‐associated steatohepatitis; R/R DLBCL, relapsed/refractory diffuse large B‐cell lymphoma.

### 4.1 Safety Strategy

Safety is an essential consideration for translating in vivo CAR‐T therapy into clinical practice. From a safety perspective, the clinical translation of in vivo CAR‐T therapies remains constrained by various major obstacles. High immunogenicity can trigger immune responses that accelerate clearance of the therapeutic vector or payload [49], while insufficient gene delivery specificity increases the potential for off‐target effects and unintended tissue damage [168]. In addition, treatment‐associated toxicities, including CRS and immune effector cell–associated neurotoxicity syndrome (ICANS), continue to constitute primary safety challenges [42]. Current strategies are focused on developing novel materials and optimizing CAR design to minimize these risks.

The immunogenicity of the delivered substance is the foremost safety consideration for in vivo CAR‐T. Both the delivery vector and the foreign genetic material it carries may elicit an immune response, leading to vector clearance and ultimately diminishing therapeutic efficacy. Immunogenicity associated with viral capsids, LNP components, and bacterial Cas9 proteins can trigger acute inflammatory responses [169], resulting in rapid vector clearance and immune‐mediated destruction of transduced cells, thereby undermining the therapeutic potential of CAR‐T cells [170, 171]. To address these concerns, strategies include the development of novel materials with improved biocompatibility and the optimization of CAR design [106, 172, 173]. For example, humanized CAR sequences and advanced gene editing tools, including high‐fidelity Cas9 variants, may be employed to reduce the immunogenicity of the CAR constructs themselves [174].

In addition to the challenge of high immunogenicity, the off‐target effects of the payload also require careful consideration [175]. The accuracy of CAR gene delivery is paramount, as the consequences of delivery errors can be difficult to predict. If the CAR gene is delivered to regulatory T cells, it could undermine anti‐tumor immunity. Conversely, if the gene is delivered to tumor cells, it could result in antigen masking, hindering CAR‐T cells’ ability to recognize the target and causing immune escape [176]. Achieving precise delivery hinges on the engineering of the vector. One strategy involves expressing T cell‐specific ligands (such as anti‐CD3e scFv) on the surface of viral vectors (e.g., lentiviruses), which significantly enhances the transduction specificity to T cells [177, 178]. Moreover, a modular system can be employed, wherein the “viral vector” and the “target head” are separated. By modifying only one end of the bispecific molecule, the virus can be redirected to different cell types without needing to re‐engineer the virus itself [179]. For non‐viral vector LNPs, targeting antibodies (such as anti‐CD5 and anti‐CD8) can be directly bound to the LNPs to achieve cell‐specific delivery [50].

After CAR‐T cells exert their antitumor effects in vivo, the potential for toxic reactions should not be overlooked. The “single injection, spontaneous expansion” nature of CAR‐T therapy may result in uncontrolled CRS and ICANS [180, 181], which, in severe cases, can be life‐threatening. To mitigate these toxic reactions, CAR design can be optimized through various strategies. The transient expression of CARs induced by LNP‐mRNA confers a natural advantage, as it allows for more controlled, short‐term CAR expression. By adjusting the dosage and frequency of administration, a “titration‐like” approach can be employed to fine‐tune CAR‐T cell activity, thereby reducing the likelihood of CRS and ICANS [55, 182]. Furthermore, small molecule drug‐controlled switches, such as SynNotch receptors, can be integrated into CAR design, or suicide genes (e.g., iCasp9) can be introduced. These platforms provide for the timely termination of CAR‐T cell activity at critical moments, thus providing an additional layer of safety control [183, 184, 185].

### 4.2 Effectiveness Strategy

Once the safety of in vivo CAR‐T therapy is ensured, the major limiting factors for its efficacy become evident: whether the generated CAR‐T cells can survive, infiltrate, and efficiently eliminate tumor cells within the TME [186]. Current strategies to overcome these challenges focus on developing multifunctional CAR‐T cells, employing co‐delivery strategies to reshape the TME, and reversing T cell exhaustion to boost their anti‐tumor efficacy [187].

The TME remains a significant obstacle for in vivo CAR‐T therapy. Tumor‐associated macrophages (TAMs) and myeloid‐derived suppressor cells (MDSCs) secrete inhibitory factors that contribute to a hypoxic and acidic environment, which impedes CAR‐T cell infiltration into the tumor and undermines their therapeutic potential [188]. To address this, co‐delivery of drugs or combination therapies are increasingly being explored to remodel the immunosuppressive TME and improve therapeutic efficacy, particularly in non‐inflamed tumors [189, 190]. For instance, A2A adenosine receptor antagonists can be co‐delivered to block the immunosuppressive signals of adenosine, reversing the immunosuppressive effects of the TME [191, 192]. Alternatively, CSF1R inhibitors targeting TAMs can be used to convert them from a pro‐tumor phenotype to a tumor‐suppressive phenotype, thereby promoting CAR‐T efficacy [193, 194]. Beyond the co‐delivery of inhibitory agents, a bicistronic Nb‐CAR‐TCE transgene can also be introduced into CD3^+^ T cells, allowing endogenous production and secretion of Nb‐BiTEs. By disrupting the PD‐1/PD‐L1 immune checkpoint axis, these secreted Nb‐BiTEs effectively reprogram the TME, thereby enhancing antitumor immune responses in solid tumors [195]. Another promising approach involves photothermal therapy (PTT), where photothermal agents are co‐delivered to degrade the tumor matrix and dilate blood vessels, facilitating better CAR‐T cell penetration into the tumor [196, 197].

The TME is a primary contributor to T cell exhaustion, which is one of the key reasons for CAR‐T cell failure and disease relapse [198]. Continuous antigen stimulation, combined with the immunosuppressive conditions of the TME, leads to the gradual loss of CAR‐T cells’ proliferative capacity and cytotoxic function. To address T cell exhaustion, multiple optimization strategies are being explored [199]. These include the integration of co‐stimulatory molecules into the CAR design for cell‐based optimization, and the co‐delivery of cytokines or immune checkpoint inhibitors to regulate CAR‐T cell efficacy. Incorporating co‐stimulatory molecules such as 4‐1BB into the intracellular signaling domain of CAR can facilitate the development of memory T cells and prolong CAR‐T cell survival [200, 201]. Moreover, co‐delivering mRNA encoding cytokines (e.g., IL‐7, IL‐15, and IL‐21) can provide vital proliferative signals to combat exhaustion and maintain CAR‐T cell functionality [202, 203, 204, 205]. In addition, the co‐delivery of immune checkpoint inhibitors, such as anti‐PD‐1 scFv mRNA, or the use of CRISPR technology to knock out inhibitory receptors (e.g., PD‐1) on CAR‐T cells, offers promising strategies to reverse the exhaustion state and improve therapeutic outcomes [174, 185]. Translating in vivo CAR‐T therapy into clinical practice is a multifaceted endeavor, relying on the fusion of innovative technologies (such as advanced vectors and rational CAR design) with a thorough understanding of biological mechanisms (e.g., overcoming TME‐induced exhaustion). This continuous interplay between technological innovation and biological insight will drive the transformation of in vivo CAR‐T from an experimental concept to a clinically applicable solution (Table 3).

**TABLE 3:** The core challenges and optimization strategies of in vivo CAR‐T therapy.

Challenge	Major barriers	Optimization strategies	References
**Targeting and delivery**			
Target specificity	Off‐target activity and systemic exposure	Precision targeting and logic‐gated designs	[46, 57, 127, 179]
Gene delivery	Inefficient in vivo cell engineering	Advanced vector and delivery technologies	[55, 57]
**CAR regulation and safety**			
CAR expression control	Imbalanced persistence and activity	Programmable expression systems	[55, 226]
Safety	Genotoxicity and immune‐mediated toxicities	Safer vector architectures and risk‐mitigation strategies	[47, 57, 226]
**Therapeutic efficacy and translation**			
Solid tumor efficacy	TME‐mediated suppression and antigen heterogeneity	Multi‐modal and multi‐target approaches	[137, 226]
Translation and manufacturing	Scalability, cost, and regulatory complexity	Standardized manufacturing platforms and regulatory harmonization	[47, 52, 57]

## 5 Conclusion and Perspective

Tumor immunotherapy has experienced significant progress, with numerous novel therapies emerging, including PD‐1 inhibitors and tumor vaccines [206, 207, 208, 209]. However, each of these approaches has inherent limitations, often resulting in suboptimal therapeutic effects. In contrast, CAR‐T therapy offers non‐MHC‐restricted targeted cytotoxicity along with the capability for prolonged persistence as a “living drug,” providing sustained immune surveillance [210]. This capability positions CAR‐T as a promising frontier in the fight against tumors. Despite its significant promise, the traditional CAR‐T manufacturing process is complex, leading to product heterogeneity and challenges in quality control. These issues contribute to low accessibility and hinder the broader clinical adoption of CAR‐T therapies.

Recently, in vivo CAR‐T has emerged as a groundbreaking alternative in the realm of tumor immunotherapy. The goal of this review is to present a comprehensive summary of the in vivo CAR‐T research landscape, beginning with an overview of the progression of CAR‐T therapy. It highlights the limitations of traditional CAR‐T production methods and underscores the merits of the in vivo CAR‐T strategy. The review also explores the various delivery vectors employed in in vivo CAR‐T therapies, encompassing both viral and non‐viral approaches. Finally, we discuss optimization strategies essential for advancing in vivo CAR‐T from laboratory innovation to clinical translation. The emergence of in vivo CAR‐T represents a pivotal milestone in cell‐based immunotherapy, transitioning from a complex and highly personalized “living drug” framework to a more efficient and accessible, gene‐therapy‐like injection model [13]. A number of in vivo CAR‐T therapies are currently undergoing clinical trials, and with further evaluation of their efficacy and safety, these therapies hold the potential to be increasingly adopted as effective tools in tumor treatment [211].

Despite its numerous advantages, the clinical translation of in vivo CAR‐T requires further optimization of manufacturing and quality control. In conventional CAR‐T strategies, the cellular product is manufactured ex vivo through a series of steps, including leukapheresis, T‐cell activation and transduction, expansion, washing and concentration, and quality‐control release testing [55, 57]. By contrast, in vivo CAR‐T shifts the cell manufacturing process into the patient, enabling CAR‐T cells to be generated in situ without extensive ex vivo culture and thereby potentially avoiding terminal differentiation before administration [52]. However, in vivo CAR‐T also faces several challenges, including the short plasma half‐life of delivery vectors, non‐specific targeting, and the predominantly quiescent state of endogenous T cells [53, 106]. Moreover, the number, phenotype, CAR expression level, and functional state of in vivo‐generated CAR‐T cells cannot be directly characterized to the same extent as those of ex vivo‐manufactured products. Therefore, dedicated monitoring and control systems are required to establish effective and safe dosing regimens, as well as to facilitate the prediction of therapeutic responses and the identification of early biomarkers of treatment‐related toxicity [107].

The transition from ex vivo to in vivo CAR‐T manufacturing necessitates extending quality control and monitoring across the entire therapeutic process. Whereas conventional CAR‐T strategies primarily focus on the quality of the final cellular product, in vivo CAR‐T requires comprehensive control of vector quality, tissue distribution, cellular targeting, transgene expression, and the characteristics of the cells generated in situ [46]. Currently, ddPCR and RNA in situ hybridization can be used to quantitatively monitor vector biodistribution and assess vector distribution across tissues, including immune organs and the liver [57]. In addition, targeted vector engineering can enable selective transduction of CD8^+^ or CD3^+^ T cells, thereby reducing off‐target transduction and hepatic transduction [53]. Effective control and monitoring systems should also incorporate indicators of patient heterogeneity. Recent studies have shown that the efficiency of in vivo CAR‐T generation can vary substantially among individuals, with a subset of mice exhibiting relatively low CAR‐T generation efficiency, potentially owing to elevated pre‐treatment levels of interferon‐α or interferon‐β, suggesting that innate immune responses can influence in vivo CAR‐T generation [106]. Future strategies may therefore require patient‐adapted dose optimization or sequential dosing regimens to achieve a more consistent therapeutic window. In addition to patient heterogeneity, the development of tunable CAR expression systems will be important. The optimal duration and magnitude of CAR expression may vary according to the disease context: durable CAR expression may be preferable for certain malignancies, whereas transient CAR expression may be sufficient for indications such as B‐cell depletion while minimizing prolonged immune activation [127]. Establishing comprehensive quality‐control and monitoring frameworks throughout the entire in vivo CAR‐T process will further enhance the advantages of this approach over conventional CAR‐T therapy and accelerate its clinical translation.

While in vivo CAR‐T therapy overcomes specific limitations of traditional CAR‐T approaches, there is still significant room for optimization. Despite notable advancements in in vivo CAR‐T delivery systems, targeting specificity remains a central hurdle. Future progress will likely rely on the integration of novel biomaterials with cutting‐edge technologies such as artificial intelligence to improve the accuracy and intelligence of delivery systems [212]. For example, machine learning algorithms can be leveraged to analyze the relationship between lipid library structures and in vivo delivery efficiency, enabling the reverse‐engineering of novel ionizable lipids that offer improved targeting capabilities and reduced toxicity [213, 214]. High‐throughput screening platforms can accelerate this process, rapidly identifying ligand‐receptor pairs that are specific to distinct T cell populations, including naive and memory T cells, thus facilitating more precise cell targeting [215, 216]. In addition, incorporating smart response elements into delivery carriers, such as “smart” LNPs or hydrogels, could enable activation by specific enzymes in the TME—for example, matrix metalloproteinases or low pH conditions. This approach would allow for targeted gene release within the TME, enhancing antitumor efficacy while reducing off‐target effects [217, 218].

As in vivo CAR‐T therapy is extended to a wider range of patients, including those with early‐stage tumors, safety becomes a critical concern. The introduction of synthetic biology concepts such as “logic gates” (e.g., AND gates, NOT gates) can provide an innovative solution by enabling CAR‐T cells to become activated only when multiple conditions—such as the coexistence of target antigens A and B—are met simultaneously [219]. This approach significantly enhances tumor specificity and reduces the likelihood of injury to normal tissues [220, 221]. In addition, integrating in vivo imaging technologies, such as reporter genes coupled with CAR genes, supports real‐time, noninvasive tracking of CAR‐T cell kinetics in vivo. This technology allows clinicians to obtain intuitive data that aids in predicting therapeutic efficacy and managing potential toxicity [222, 223]. Looking ahead, in vivo CAR‐T therapy has applications beyond tumor treatment. In non‐tumor contexts, transient CAR‐T cells can be employed to precisely target and eliminate pathogenic cells, offering new therapeutic strategies for autoimmune diseases (e.g., systemic lupus erythematosus, multiple sclerosis), fibrotic diseases (e.g., cardiac, liver, and lung fibrosis), and even chronic viral infections (e.g., HIV, hepatitis B) [65, 137, 224]. Future research should focus on advancing technologies to improve the intelligence, safety, and effectiveness of in vivo CAR‐T delivery vectors, further broadening their clinical applications. In conclusion, in vivo CAR‐T holds great potential by optimizing traditional CAR‐T strategies, simplifying procedures, reducing costs, improving cell quality, and enabling more flexible, controllable treatments. Continued improvements in safety and efficacy will accelerate its clinical adoption and play a pivotal role in advancing tumor immunotherapy.

## Author Contributions

Z.B. performed data curation, wrote the original draft, and conducted the literature search. M.Y. wrote the original draft, performed visualization, and conducted selection. S.H. performed visualization and screening. D.X. conducted the literature search. L.W. performed writing, review, and editing. L.Z. created the conceptualization, review, methodology, and supervision. Z.D. created the conceptualization, acquired funding, performed writing, review, editing, visualization, and supervision, and provided resources. All authors have read and approved the final manuscript.

## Funding

This work was supported by the National Natural Science Foundation of China (Grant Nos. 82573524, 82503827, and 82373281), the Natural Science Foundation of Zhejiang Province (Grant No. LQ24H160007), and the Suzhou Science and Technology Program Project of 2025 (Grant No. szm2025001).

## Ethics Statement

The authors have nothing to report.

## Conflicts of Interest

The authors declare no conflicts of interest.

## Supporting information




**Supporting File 1**: mco271030‐sup‐0001‐SuppMat.docx

## Data Availability

The authors have nothing to report.
