# Supplementary material for: Advancing In Vivo Chimeric Antigen Receptor T‐Cell Engineering to Accelerate Clinical Translation
Source: MedComm (2020). 2026 Sep 27;7(10):e71030. doi: 10.1002/mco2.71030 (PMC13616186; doi:10.1002/mco2.71030)
Supplement: Supplementary file 1 [file MCO2-7-e71030-s001.docx]

**Advancing in vivo Chimeric Antigen Receptor T-Cell Engineering to Accelerate Clinical Translation**

Zhenxin Bai^1,2,#^, Ming Yi^2,#^, Shengtao Hu^2,#^, Dixuan Xue^2^, Luxin Wei^1^, Lifeng Zhang^1,*^, Zhijun Dai^1,2,*^

1. Department of general surgery, The Fourth Affiliated Hospital of Soochow University (Suzhou Dushu Lake Hospital), Suzhou 215000, Jiangsu, China.

2. Department of Breast Surgery, First Affiliated Hospital, School of Medicine, Zhejiang University, Hangzhou 310003, China.

^*^Address correspondence to: Zhijun Dai; [dzj0911@126.com](mailto:dzj0911@126.com) and Lifeng Zhang; [zhanglifeng@suda.edu.cn](mailto:zhanglifeng@suda.edu.cn)

^#^These authors contributed equally to this work.

Supplementary Materials

***Table S1*.** Comparison of viral and non-viral vector platforms for in vivo CAR-T cell engineering.

| Dimension | Viral vectors (LV / AAV) | Non-viral vectors (LNP / Polymer) | References |
| --- | --- | --- | --- |
| Gene delivery and persistence | High transduction efficiency with durable transgene expression; sustained expansion supported by persistent antigen stimulation | Moderate delivery efficiency with generally transient expression; prolonged expression achievable using non-integrating DNA platforms | ^47,55,57,103^ |
| Safety Profile | Risk of insertional mutagenesis (LV) and vector-associated immunogenicity; potential risks of severe or prolonged CRS and ICANS | Low genotoxicity risk owing to non-integrating delivery; lower immunogenicity and improved safety through transient and dose-adjustable expression | ^55,65,85,91,106,137^ |
| Manufacturing and scalability | Complex manufacturing workflow, high production costs, and limited scalability | Scalable and standardized manufacturing with lower costs and potential for off-the-shelf deployment | ^47,57,103^ |
| Clinical translation and regulatory considerations | Extensive clinical validation with established proof-of-concept; requires long-term safety monitoring for genotoxicity | Early-stage clinical development; enables pharmacological control through repeat dosing and faces fewer genotoxicity-related regulatory constraints | ^47,57^ |
